# Can we predict vascular status and culture result based through wound status in diabetic foot infection?

**DOI:** 10.1097/MD.0000000000014892

**Published:** 2019-03-22

**Authors:** Jin Yong Shin, Si-Gyun Roh, Suk Choo Chang, Nae-Ho Lee

**Affiliations:** a Department of Plastic and Reconstructive Surgery, Medical School of Chonbuk National University; b Research Institute of Clinical Medicine of Chonbuk National University-Biomedical Research Institute of Chonbuk National University Hospital, Jeonju, Republic of Korea.

**Keywords:** culture results, diabetic foot, diabetic foot ulcer, vascular status

## Abstract

Diabetic foot infection (DFI) should be treated by a multidisciplinary team to prevent amputation and morbid status. As physicians encountering DFI in outpatient clinic, a proper selection of antibiotic treatment and diagnostic approach for a vascular status is essential. We retrospectively investigated the patients with DFI from 2016 to 2017. All patients were examined for vascular status, wound status, and pathologic culture preceding the treatment. No statistical significance was observed between PEDIS grade 1 and 2 and 3 and 4 in culture status and culture results. Association analysis between vascular status and other variables, such as wound score and culture results, has no significant difference. Through these results, the helpful epidemiologic result of microbiology and necessity of examination for peripheral arterial disease were verified.

## 1 Introduction

Diabetic foot ulcers have a high possibility of infection that can spread rapidly, leading to amputation.^[1]^ Lower limb amputation in patients with diabetic foot accounts for 50% of nontraumatic amputations of the lower leg.^[2]^ For preventing amputation in patients with diabetic foot infection (DFI), proper diagnostic and treatment approach should be performed and classified by severity. Clinicians should make decisions on which patients must be hospitalized and undergo surgical debridement.^[3]^ Empiric antibiotic treatment should be also chosen according to clinical and epidemiologic data and other multi-disciplinary approaches must be performed such as medical assistance for comorbid disease such as peripheral arterial disease (PAD).

Proper selection of antibiotic regimen is an essential treatment for DFI. Gram-positive cocci in diabetic foot ulcer are the most common pathogens. However, according to severity of infection state, empiric antibiotic treatment is more important.^[4,5]^ The epidemiologic characteristic of microbiology should be also considered.^[6]^ Besides antibiotic treatment, the PAD is also frequently present in patients with diabetic foot up to 50%.^[7]^ PAD was also investigated as a main factor of poor prognosis.^[8]^ Proper revascularization for PAD has been studied and it has several advantages, such as improved vascularity and wound healing, in patients of diabetic foot.^[9]^

This study planned to verify that wound status in first encountered state of outpatient clinic predict the kinds of micropathogens and vascular status in patients with DFI. If this hypothesis is verified, the efficient empiric antibiotic treatment will be possible and necessity of examination and treatment of vascular problem will be explained well to patients.

## 2 Method

Patients with DFI from 2016 to 2017 were investigated retrospectively. The result of each test and epidemiological characteristics were checked through medical charts. All patients were examined for vascular status, wound status, and pathologic culture preceding treatment.

Lower computed tomographic angiography (CTA) was usually performed for testing of vascular status and patients with exceptional kidney disease were tested by Doppler ultrasonography. The results were divided with normal and abnormal, noting one or more vessels have problems. The vessel that has clogging was marked. When complications in vessels arise, the confirmative percutaneous intraluminal angiography was performed and the vessels were revascularized.

The angiosome concept is a unit of tissue supplied by a source artery. It has 3-dimensional network of vessels.^[10]^ The wound of the diabetic foot was examined physically and evaluated with wound score (DFI wound score) suggested by Infections Disease Society of America (IDSA) and PEDIS grade.^[11]^ To evaluate wound, this 10-item scoring system was developed. The PEDIS grade was classified from 1 to 4 according to wound infection. PEDIS grade 1 and 2 state that uninfected or mild infection in diabetic foot. PEDIS grade 3 and 4 state that moderate and severe infection in diabetic foot.

Wound culture was performed with deep tissue after surgical debridement.

The association between wound status, vascular status and culture results were verified by Fisher exact test or Chi-squared test. R language version 3.3.3 (R Foundation for Statistical Computing, Vienna, Austria) and T&F program version 2.5 (YooJin BioSoft, Korea) were used for all statistical analyses

This study was approved by the institutional review board.

## 3 Result

Epidemiologic characteristics of patients such as sex, age and wound score, vascular status, and culture results were shown in Table 1. There were 3 times more men than women and the average age was 60.48 years old. The ratio between pedis grade 1,2 and 3,4 was 1:1. The normal vascular status was shown in 65.5% of patients and multifocal stenosis of vessels was the most when there is stenosis of vessels. In the result of culture, gram-positive and negative aerobic bacteria accounted for most of them.

**Table 1:**
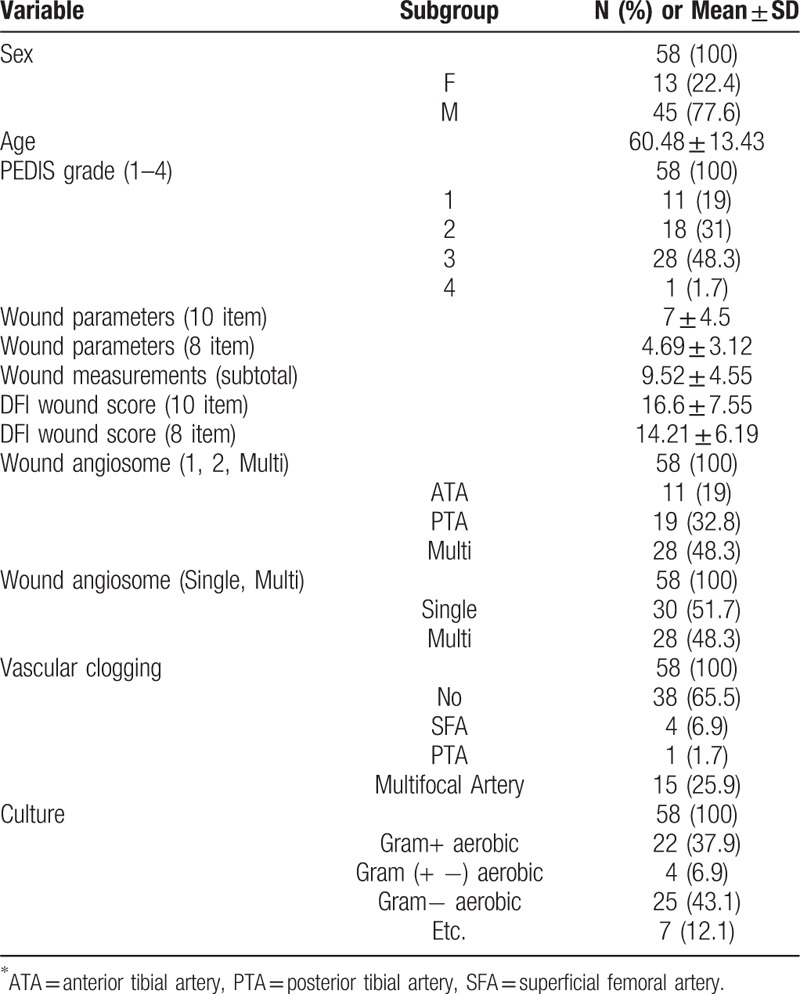
Baseline characteristics of variables.

There is no statistical significance between PEDIS grade 1,2 and 3,4 in wound status and culture results (Table 2). There is also no correlation between group of 10 items DFI wound score less than 20 and DFI wound score more than 20 (Table 3). We found that gram-negative aerobic bacteria were cultured significantly in the group of 8 items DFI wound score of more than 25 (Table 4). Association analysis between vascular status and other variables like wound score and culture results has no significant difference (Table 5).

**Table 2:**
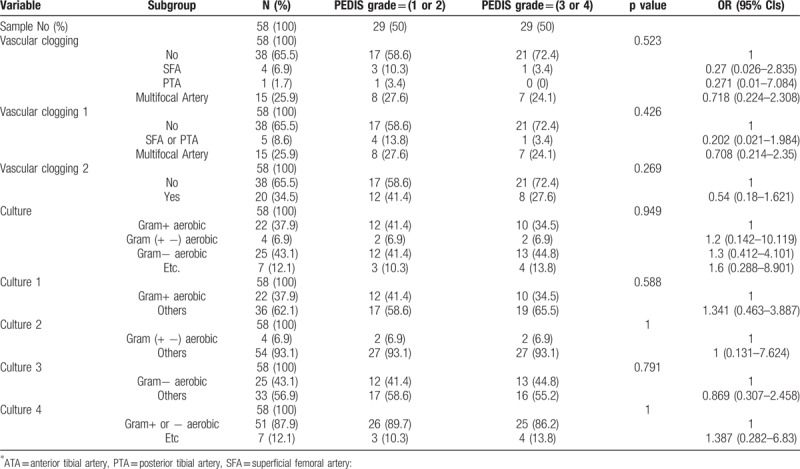
Association analysis between PEDIS grade (1,2 vs 3,4) and categorical variables using contingency table.

**Table 3:**
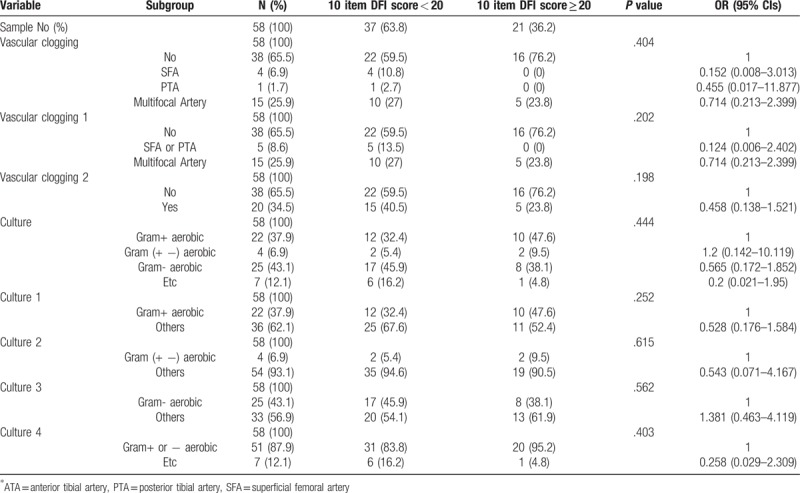
Association analysis between 10 item DFI score (cutoff = 20) and categorical variables using contingency table.

**Table 4:**
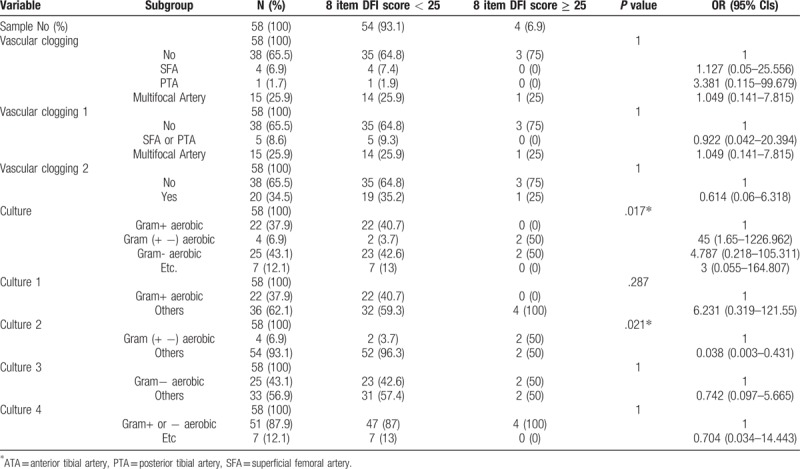
Association analysis between 8 item score (cutoff = 25) and categorical variables using contingency table.

**Table 5:**
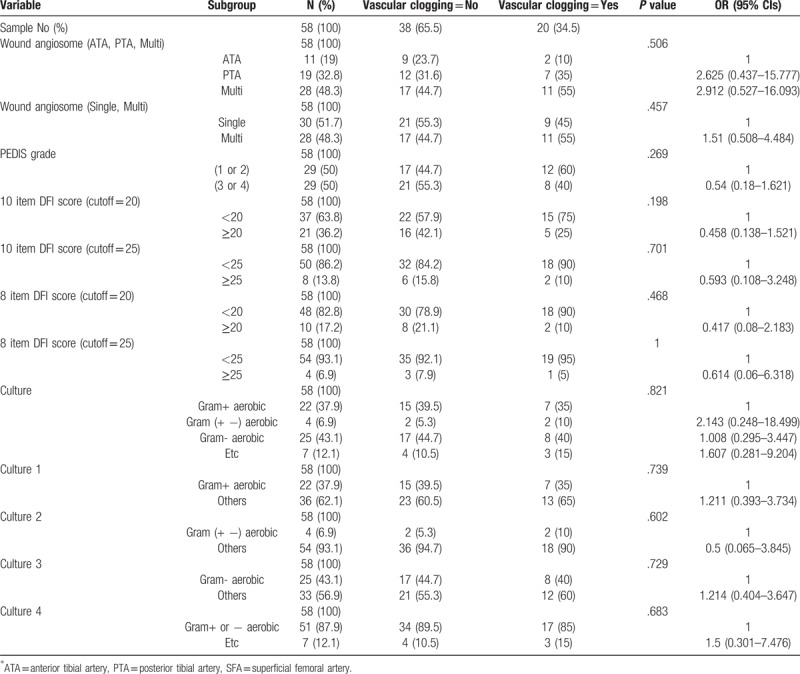
Association analysis between vascular clogging and categorical variables using contingency table.

## 4 Discussion

DFI requires careful management and a multidisciplinary foot-care team. Without proper understanding of this disease, it leads to medical morbidity, amputation, financial costs, and more hospital days.^[12,13]^ Besides medical cooperation, this study concentrated on the microbiology and vascular status in DFI. If we are able to use microbiology and vascular status through initial wound assessment, more effective and proper management of infections will be possible.

Microbiology in DFI was already researched globally in many previous studies. The most commonly isolated pathogens are aerobic gram-positive cocci. In chronic and complex wounds, the variable gram-negative rods or anaerobes could be colonized.^[2,11,14]^ However, there are diverse characteristic of cultured pathogen depending on region, social and economic level. Empirical antibiotic treatment previous culture result should follow regional characteristics of microbiology.^[15–17]^ In our single center study, the commonly cultured pathogens were gram-positive cocci and in a little severe wound, gram-negative bacteria were also cultured. (Table 4) Even so, severe wound status more than DFI wound score 25 was so few (6.9%) that this result was most likely false positive. Due to improved sanitary conditions and awareness development, the moderate to severe foot infection were too few to analyze quantitatively.

PAD is also a common comorbid disease in patients with diabetic foot. Some previous study showed up to 50% of PADin diabetic foot patients^[7]^ and even cardiovascular disease was also present with high possibility in addition to PAD.^[18]^ About 35% of our investigated patients were found to be have at least 1 arterial problem below knee and more than half of those were found to be multifocal arterial occlusion. Unlike our hypothesis, there are no significant relationships between vascular status and wound status (Table 5). These results support that diagnostic test for vascular occlusion should be performed in diabetic foot patients regardless of the wound status.

As mentioned above, the encountered patients with DFI in clinic can be guided and treated in terms of the wound status, vascular status, and microbiology. First, unconditional diagnostic test for vascular status should be progressed following result that more than 30% of all patients with DFI had vessel occlusion below knee. If one or more vessel occlusion below the knee is found, angioplasty or vascular surgery can be recommended for better wound healing.^[9,19,20]^ Secondly, although microbiologic analysis could not be possible due to inadequacy of severe wound infection, there are almost gram-positive bacteria cultured so that the target for antibiotic treatment should be determined. Of course, special situation for choosing empiric antibiotic treatment should be considered, such as recently received antibiotic treatment, severe infection or prior history of methicillin-resistant *Staphylococcus aureus*, etc.^[11,21,22]^

Wound assessment by using wound scale is very useful for determining wound status and treatment modality. However, only wound status was not enough to assess vascular status and microbiology in patients of DFI. The empiric antibiotic targeting gram-positive bacteria should be chosen before culture result in the majority of cases. There are also other various situations needing consideration for selection of special antibiotics in treatment of gram-negative or anaerobic bacteria. Another problem of diabetic foot is that vascular occlusion should be also assessed in all patients with DFI. The necessity of revascularization must be appraised by physical examination and possible diagnostic tests.

## Author contributions

**Conceptualization:** Nae-Ho Lee.

**Data curation:** Nae-Ho Lee.

**Formal analysis:** Jin Yong Shin.

**Funding acquisition:** Jin Yong Shin.

**Investigation:** Si-Gyun Roh.

**Methodology:** Jin Yong Shin, Si-Gyun Roh, Suk Choo Chang.

**Resources:** Jin Yong Shin, Nae-Ho Lee.

**Software:** Si-Gyun Roh, Suk Choo Chang.

**Supervision:** Suk Choo Chang, Nae-Ho Lee.

**Validation:** Suk Choo Chang, Nae-Ho Lee.

**Visualization:** Nae-Ho Lee.

Nae-Ho Lee orcid: 0000-0003-1354-8203.
